# Optimization and Characterization of Chitosan Films for Transdermal Delivery of Ondansetron

**DOI:** 10.3390/molecules18055455

**Published:** 2013-05-10

**Authors:** Aslı Sedef Can, Meryem Sedef Erdal, Sevgi Güngör, Yıldız Özsoy

**Affiliations:** Department of Pharmaceutical Technology, Faculty of Pharmacy, Istanbul University, Istanbul 34116, Turkey; E-Mails: cansedef@yahoo.com (A.S.C.); serdal@istanbul.edu.tr (M.S.E.); sgungor@istanbul.edu.tr (S.G.)

**Keywords:** chitosan, ondansetron hydrochloride, terpenes, transcutol, transdermal film

## Abstract

The aim of this study was to develop novel transdermal films of ondansetron HCl with high molecular weight chitosan as matrix polymer and 2-(2-ethoxy-ethoxy) ethanol (Transcutol^®^) as plasticizer. In this context, firstly the physicochemical properties of gels used to formulate transdermal films were characterized and, physicochemical properties and bioadhesiveness of the transdermal films prepared with chitosan gels were assessed. The impact of three different types of terpenes, namely limonene, nerolidol and eucalyptol on *in vitro* skin permeation of ondansetron from transdermal films were also examined. ATR-FTIR measurements were performed to investigate the effects of the chitosan film formulations on *in vitro* conformational order of *stratum corneum* intercellular lipids after 24 h permeation study. The results showed that the chitosan gels consisting of Transcutol^®^ as plasticizer and terpenes as penetration enhancer may be used to prepare transdermal films of ondansetron due to the good mechanical properties and bioadhesiveness of the transdermal films. Eucalyptol (1%) showed higher permeation enhancer effect than the other terpenes and control. ATR-FTIR data confirmed that finding in which eucalyptol induced a blue shift in the both CH_2_ asymmetric and symmetric absorbance peak positions indicating increased lipid fluidity of *stratum corneum*.

## 1. Introduction

Transdermal therapeutic systems, also defined as “patches”, deliver therapeutically effective amounts of drugs to the systemic circulation via skin. Transdermal systems have numerous superiorities over oral dosage forms such as overcoming first-pass metabolism, improved patient compliance, and reduced gastrointestinal side effects [1,2]. Polymers are the most important components of these systems in terms of release and permeation characteristics of drugs as well as mechanical properties of the formulations. Besides, plasticizers and permeation enhancers have considerable effect on the permeability, wearing properties formulation of transdermal patches [3,4,5]. Several studies have been performed in which natural polymers used as matrix agent to optimize transdermal systems [6,7].

Chitosan is a natural polycationic polysaccharide which is used in various pharmaceutical drug delivery systems due to its favorable properties such as biocompatibility, nontoxicity, biodegradability and gel forming ability [8,9,10]. Chitosan is able to enhance the paracellular permeability of mucosal membranes by opening the tight junctions and thereby improving the penetration of drug compounds [3]. There are also studies in which chitosan is used as a biopolymer controlling the release rate of the drug in transdermal drug delivery systems and the bioadhesive property of chitosan has great impact regarding both dermal and transdermal applications [8,11,12,13,14]. It has been proven that in *stratum corneum* the tight junctions between cells has negative charges similar to those found in epithelial cells. Therefore the bioadhesive property as well as the percutaneous penetration enhancement activity of chitosan can be attributed to the interaction between its positive charges and negative charges of skin [15,16]. The physical properties of chitosan based transdermal films such as elasticity; fragility and duration have been shown to be favorable [8,14]. 

Ondansetron hydrochloride (OND) is a selective 5-HT_3_ receptor antagonist used in the treatment of nausea and vomiting related to cancer chemotherapy [17]. Due to its relatively low oral bioavailability and the patient inconvenience of intravenous administration, transdermal delivery of OND could be an alternative delivery approach. As a molecule based on its physicochemical properties, OND appears to be a suitable transdermal agent. It has a molecular weight of 293 Da, a logP value of 2.07 and its pKa is about 7.4 [18,19]. The effects of various vehicles, delivery systems and enhancers on transdermal OND delivery have been reported in the literature [19,20,21,22], but there are a few reports indicating the possibility of developing transdermal film formulations of OND [22,23,24,25].

The present study was intended to investigate the potential of chitosan based films for the transdermal delivery of OND. In this perspective the physicochemical, mechanical and bioadhesive properties of the developed formulations were examined and the effect of some terpene penetration enhancers on *in vitro* OND permeation through dermatomed pig skin was investigated. The skin interaction of the enhancers on a molecular level was evaluated by ATR-FTIR spectroscopy.

## 2. Results and Discussion

**Figure 1:**
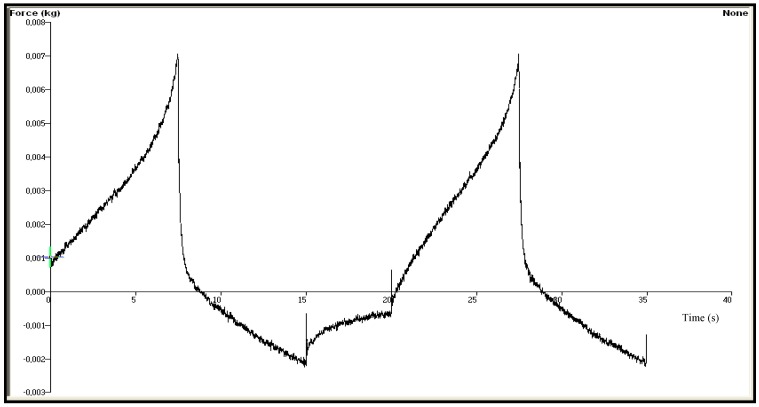
The force-time plot of chitosan based gel formulation G-LIMO containing limonene as penetration enhancer.

**Figure 2:**
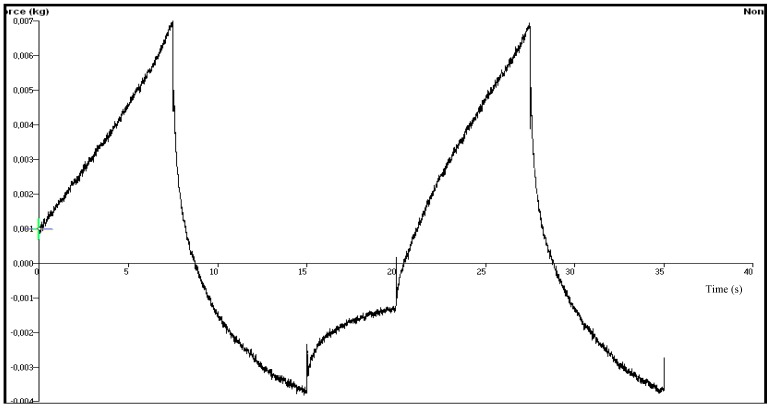
The force-time plot of chitosan based gel formulation G-NERO containing nerolidol as penetration enhancer.

**Figure 3:**
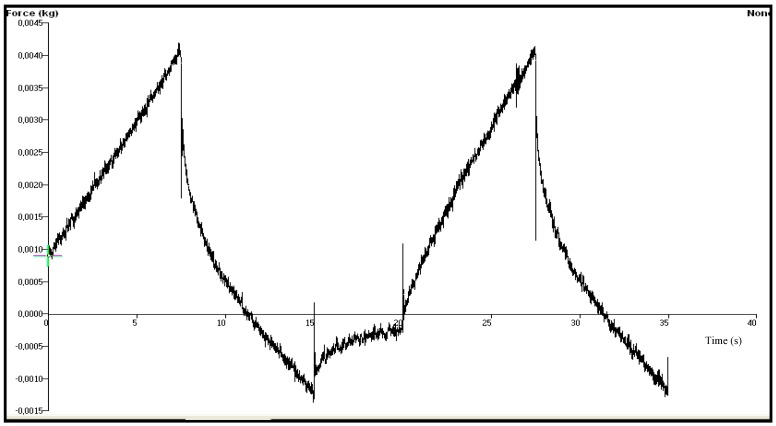
The force-time plot of chitosan based gel formulation G-EUCO containing eucalyptol as penetration enhancer.

In our study, chitosan based gel formulations of OND were prepared firstly and three different type of terpenes, namely limonene, nerolidol, or eucalyptol, were incorporated into the formulations as penetration enhancers. The difference in the mechanical characteristics of the gels was investigated using texture profile analyzer (TPA). TPA has been reported as a simple and rapid technique which provides information about the mechanical parameters of a gel formulation such as hardness, adhesiveness, compressibility, cohesiveness and elasticity [26]. These parameters have direct relevance to the performance of products for transdermal application. The force-time plots of chitosan based gel formulations coded with G-LIMO, G-NERO and G-EUCO used for the determination of the mechanical properties are shown in Figure 1, Figure 2 and Figure 3 and the data obtained from those force-time plots is summarized in Table 1.

**Table 1:** The mechanical properties of chitosan based gel formulations G-LIMO, G-NERO and G-EUCO used to formulate transdermal films of OND (mean ± SD, n = 6).

Chitosan Gels	Hardness (N)	Elasticity	Cohesiveness	Adhesiveness (µJ)	Compressiblity (N.mm)
G-LIMO	0.059 ± 0.006	0.904 ± 0.078	0.956 ± 0.046	123.789 ± 10.091	0.023 ± 0.004
G-NERO	0.040 ± 0.002	0.956 ± 0.098	0.909 ± 0.078	120.345 ± 8.963	0.022 ± 0.003
G-EUCO	0.069 ± 0.005	0.978 ± 0.092	0.933 ± 0.089	117.357 ± 9.089	0.030 ± 0.007

The hardness parameter which is related to the strength of the gel structure under compression introduces the required force to provide the deformation of a gel formulation. It expresses the applicability of the gel to the desired site. That parameter significantly affects both pourability and spreadability of the gel into film molds. The highest force value in the first immersion period of the probe gives the hardness value required to the deformation of the gel [7,27,28]. A low hardness value has been reported as an advantage for dermal or mucosal application of formulations [28,29,30]. Our results showed that the gel formulation G-NERO had the lowest hardness value at body temperature.

Elasticity is defined as the direction of reconstruction of the gel after its deformation by compression in the means of time. The ratio of the time required to produce the highest deformation in the second and first immersion period shows the elasticity of the gels. Elasticity of the gels would have great impact on the elasticity of the transdermal films prepared with the gels. The increase in the quantitative value of elasticity obtained during TPA shows the decrease in the elasticity of the gel formulation [31]. We observed acceptable elasticity results, and eucalyptol-containing gel formulation showed the highest elasticity value (0.978 ± 0.092). This result in accordance with a previous study, where a sodium alginate based gel of metoclopramide containing eucalyptol (1%) as penetration enhancer showed the highest elasticity value among the formulations containing limonene, nerolidol and terpinolene [7].

Cohesiveness is a measure of the degree of difficulty in breaking down the gels internal structure [27]. The ratio of the area under the first and second immersion gives the cohesiveness value of the gels. Cohesiveness parameter of the gels has considerable impact on the strength and the elasticity of transdermal films. The high cohesiveness values of the chitosan based gels prepared in this study are indicative of the ability to prepare homogeneous films. There was not a significant difference between the gels (*p* > 0.05) in the mean of cohesiveness indicating that the type of the penetration enhancer did not affect cohesiveness of the gels. 

Adhesiveness represents the work required to overcome the attractive forces between the surface of the gel and the surface of the probe and calculated from the negative force area obtained during the first immersion period [30,31]. In our study the highest adhesiveness value was obtained with the gel formulation G-LIMO, followed by G-NERO and G-EUCO, but the difference was statistically insignificant (*p* > 0.05). 

The compressibility defines the required work for the compaction of the gel along a definite distance [31,32]. According to our TPA data it can be assumed that the spreadability of G-LIMO and G-NERO can be better than that of G-EUCO because of their low compressibility value. 

It was evident from the TPA data that all of the chitosan based gel formulations showed suitable mechanical characteristics and based on that data it was concluded that the chitosan gels consisting of Transcutol^®^ P and a terpene as plasticizer and penetration enhancer, respectively could be used to prepare the transdermal films of OND. For a first assessment of the suitability of the films for topical application on skin, organoleptic evaluations were performed. The transdermal films of OND prepared in this study coded with T-CONT, T-LIMO, T-NERO, and T-EUCO were slightly opaque, smooth and flexible. The thickness, weight and drug content of the film formulations are represented in Table 2. 

**Table 2:** Thickness, weight and drug content uniformity of the transdermal films (mean ± SD, n = 3–6).

Transdermal Film	Thickness (mm)	Weight (mg.cm^−2^)	OND Content (%)
T-CONT	0.10 ± 0.03	33.57 ± 1.62	97.13 ± 0.23
T-LIMO	0.12 ± 0.02	35.23 ±1.54	96.92 ± 0.41
T-NERO	0.10 ± 0.02	34.56 ± 1.23	97.15 ± 1.56
T-EUCO	0.15 ± 0.04	36.45 ± 1.45	97.89 ± 0.63

The thickness of OND transdermal films was found between 0.10 ± 0.03 and 0.15 ± 0.04 mm and the standard deviation of weight was less than 2%, indicating homogeneity of the films. The chitosan films were generally thin and therefore aesthetically acceptable. The OND content of the films was between 96.92 ± 0.41% and 97.89 ± 0.63%, confirming the reproducibility of the manufacturing process. Estimation of OND content at different parts of transdermal film formulations indicated that the drug is uniformly distributed throughout the films. The content uniformity of all formulations satisfied pharmacopeia requirements for transdermal delivery systems evidenced by the low standard deviation values. This denotes also that the rheological and mechanical properties of the chitosan gel formulations were suitable to provide homogeneous drug distribution throughout the casting and drying process.

**Figure 4:**
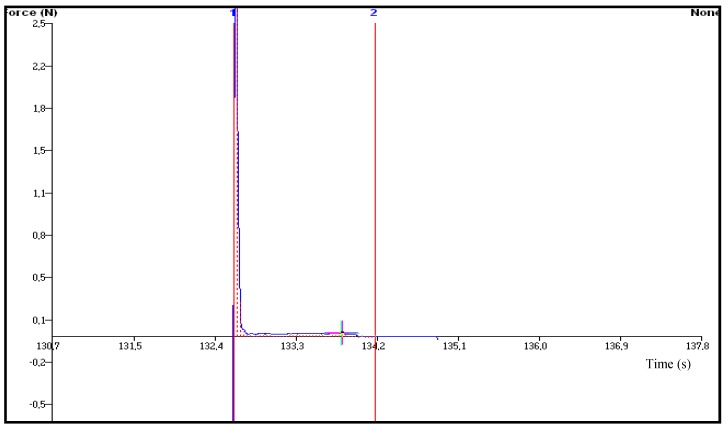
Bioadhesion force plot of T-CONT.

The bioadhesion of transdermal delivery systems is a critical factor directly related to drug delivery and therapeutic effect which in turn is the sign of product safety, efficiency and quality [33,34]. In matrix type transdermal systems where the drug is dispersed or solubilised in a polymer matrix, the quality of contact between the film and the skin determines the consistency of drug delivery through skin. Therefore we evaluated the *in vitro* bioadhesion of the prepared OND film based on chitosan polymer matrix. The *in vitro* bioadhesion force value of T-CONT was 1.776 ± 0.16 µJ/mm^2^ indicating a sufficient adhesiveness to skin (n = 3) (Figure 4). It was shown that the adhesion of thicker films to porcine skin was significantly lower *in vitro* than thinner films [35]. The thickness value of T-CONT was 0.10 ± 0.03 and as expected a satisfactory bioadhesion value was obtained with this thin film. 

The choice of the polymer and a suitable plasticizer are the most important parameters for the development of the polymeric films [15]. In previous studies the plasticizing effect of various hydrophilic and lipophilic solvents, *i.e.*, water, sorbitol, sucrose, glycerine, propylene glycol, triacetin, di-*n*-butyl phthalate and polyethylene glycol 400, on chitosan or chitosan-synthetic polymer blends have been investigated [15,36,37]. In pre-formulation studies, we observed that the unplasticised chitosan films were nonflexible and brittle (*data not given*). Correspondingly, the use of Transcutol^®^ P as plasticizer improved the mechanical properties of the developed films in terms of flexibility and elasticity. During the *in vitro* bioadhesion test, the film formulation T-CONT was stripped cleanly from the skin and left no visually residue. Transdermal films are expected to adhere spontaneously to the surface they contact and upon removal they have to leave no adhesive residue. 

**Figure 5:**
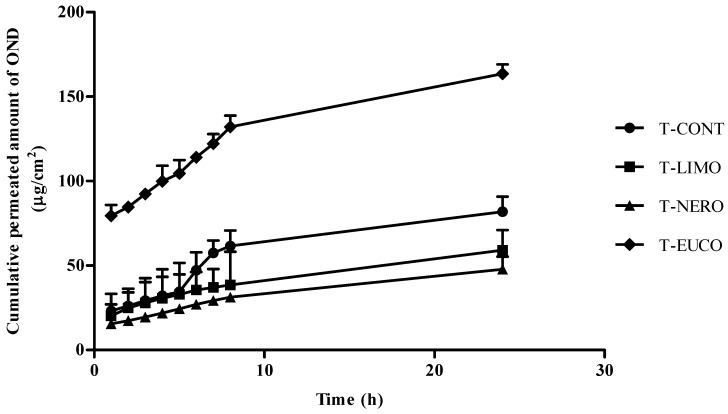
*In vitro* permeation of OND from chitosan based transdermal film formulations across pig skin.

The addition of penetration enhancers to topical or transdermal delivery systems may improve drug permeation through the skin either by altering the skin barrier or by modifying the thermodynamic activity of penetrants [5]. Terpenes are safe and effective penetration enhancers, classified as generally regarded as safe (GRAS) by FDA. They have been found as efficient penetration enhancers for drugs with differing lipophilicities. Terpenes were reported to cause no or minimal skin irritancy and they have reversible effects on skin lipids [20,38,39,40,41]. We studied the effect of three different types of terpenes including limonene (a hydrocarbon lipophilic monoterpene), nerolidol (an amphiphilic sesquiterpene) and eucalyptol (an oxygen containing monoterpene) on *in vitro* permeation characteristics of OND from chitosan transdermal films across pig skin. The permeation profiles observed are given in Figure 5 and Table 3 reports flux (J_ss_), enhancement ratios for flux (ER_flux_) and the cumulative corrected receptor concentrations at 24 h (Q_24_) from this study.

**Table 3:** Effect of chemical enhancers on skin permeation of OND from chitosan based transdermal film formulations (mean ± D, n = 6).

Transdermal Film	Flux (*J_ss_*) (μg·cm^−2^·h^−1^)	ER_Flux_	Q_24_ (μg·cm^−2^)
T-CONT	2.81 ± 2.56	1.00	81.93
T-LIMO	2.64 ± 2.45	0.94	59.1
T-NERO	2.38 ± 3.64	0.85	47.77
T-EUCO	9.07 ± 4.34	3.23	163.59

As can be seen from the Figure 5, the permeation rate of OND from the chitosan film (T-EUCO) containing eucalyptol as penetration enhancer was significantly higher than those of other two formulations containing either nerolidol or limonene and control formulation (T-CONT) (*p* < 0.05). The highest OND flux (9.07 ± 4.34 µg/cm^2^/h^−1^) across pig skin was observed with T-EUCO and the enhancement ratio of that transdermal film was found to be about 3.2. However, the addition of limonene and nerolidol to the chitosan films as enhancer was not lead to significant increase in OND flux across skin compared to control formulation (*p* > 0.05) and cumulative amount permeated OND from transdermal films containing either nerolidol (T-NERO) or limonene (T-LIMO) showed was lower than those of T-CONT and T-EUCO. That decrease can be attributed to the decreased partitioning of OND into the skin from vehicle in the presence of the very lipophilic terpenes such as limonene and nerolidol. Eucalyptol is a moderately lipophilic terpene with a log P value of 2.74. Conversely, the lipophilicity of limonene and nerolidol as donated by log P is 4.58 and 5.36, respectively [38]. Krisnaiah *et al.* showed that OND flux increased from the hydroxypropyl cellulose gel drug reservoir systems consisting of nerolidol or limonene as enhancer. However, that permeation study was performed on rat epidermis which have more numbers of hair follicle and permeable than that of pig skin [21]. Therefore, the discrepancy on OND permeability of nerolidol and limonene can be due to the difference on skin species. But, it should be also taken into account that pig skin is more acceptable skin model possessing quite similar permeability properties to human skin [42]. 

Thermodynamic activity of terpenes as well as log P, and boiling point also play a major role in enhancing the permeability of drug compounds. In literature, the increase in concentrations of terpenes (10%) was resulted in high permeation enhancement of propranolol HCl [36] and the greatest enhancement in skin absorption of diclofenac sodium was observed with the highest concentration of terpenes (2.5%) [43]. The differences in penetration enhancing activity at lower concentrations than 5% have been explained by the fact that the differences among the thermodynamic activity of terpenes in vehicle [44]. However, in our study terpenes could not be incorporated into the chitosan gel matrix at more than 1% concentration in spite of adding Tween 80 as emulsifying agent into the formulation. Therefore, the retarding effect of nerolidol and limonene on skin permeation of OND from chitosan transdermal films may be due to the lower thermodynamic activity of terpenes in the formulation. However, further investigations are required to explain this effect.

**Figure 6:**
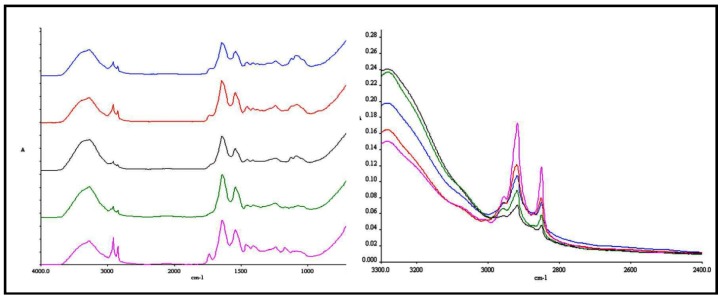
ATR-FTIR spectra of skin before and after treatment with chitosan transdermal films of OND (Pink: Control skin, Green: Skin treated with T-CONT, Black: Skin treated with T-EUCO, Red: Skin treated with T-NERO and Blue: Skin treated with T-LIMO).

**Figure 7:**
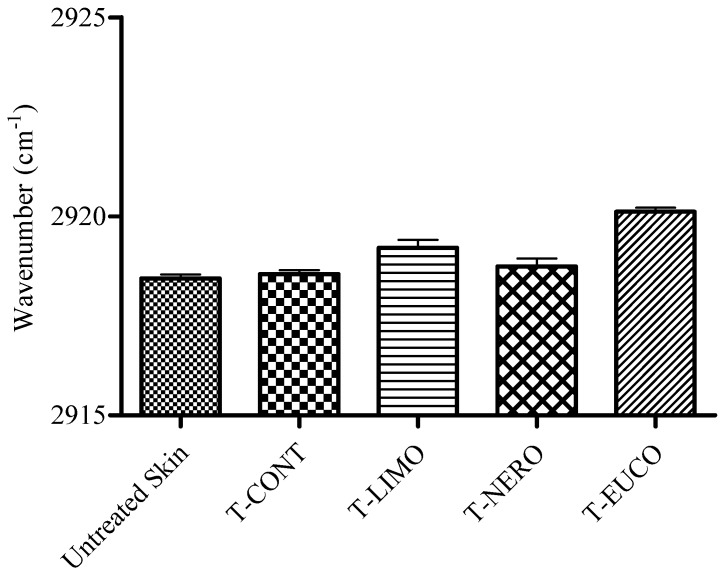
Peak positions of skin lipids CH_2_ asymmetric stretching absorbances after application of OND transdermal films.

**Figure 8:**
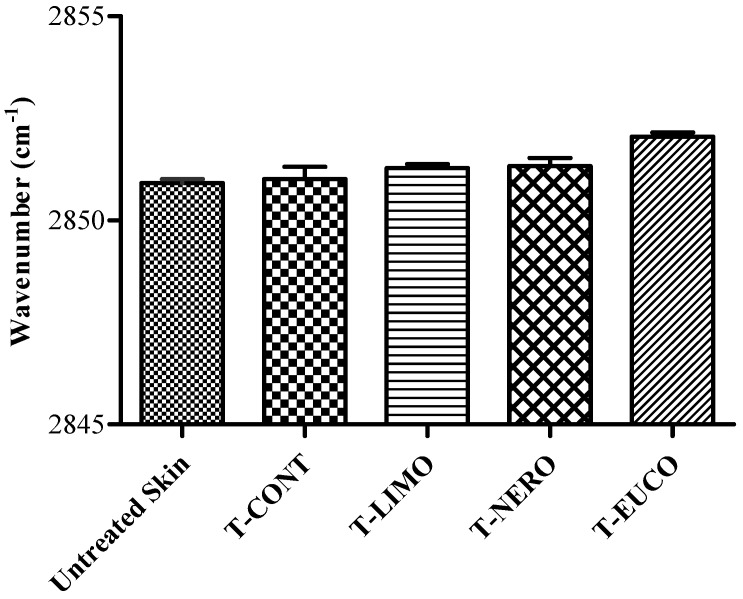
Peak positions of skin lipids CH_2_ symmetric stretching absorbances after application of OND transdermal films.

To investigate the effects of the chitosan film formulations on *in vitro* conformational order of *stratum corneum* intercellular lipids following permeation study for 24 h, ATR-FTIR measurements were conducted on skin spices. The IR spectra of skin before and after application of transdermal films are shown in Figure 6. The absorbance frequency shift of CH_2_ asymmetric and symmetric absorbance peaks originating from the hydrophobic alkyl chain of *stratum corneum* lipids was analyzed and the results are given in Figure 7 and Figure 8.

It was reported that oxygen containing terpenes like eucalyptol exert their enhancing effect both upon introducing a disorder in lipid alkyl chains in *stratum corneum* and by disruption the hydrogen bond network of the lipid bilayer [41,45,46]. Our ATR-FTIR results showed a more disordered *stratum corneum* lipid state in the presence of eucalyptol as enhancer. T-EUCO induced a blue shift in the both CH_2_ asymmetric and symmetric absorbance peak positions by 2 cm^−1^ indicating increased lipid fluidity which is in turn a decrease in diffusional resistance to permeant. This result is in agreement with our *in vitro* skin permeation data, where the film formulation T-EUCO showed the highest drug flux for OND. The permeation effect of eucalyptol could involve its distribution into the lipid domain of *stratum corneum* and the reversible disruption of the intercellular lipids.

## 3. Experimental

### 3.1. Materials

Ondansetron hydrochloride (OND) was kindly provided from Nobel Drug Company (Istanbul, Turkey). Chitosan (high molecular weight [MW: 140,000–220,000], % deacetylation > 75), limonene, nerolidol, and eucalyptol were purchased from Sigma Aldrich (Steinheim, Germany). Transcutol^®^ P was a gift from Gattefossé (Cedex, France). Deionized water was employed from an ELGA Purelab Ultra purification system (Marlow, UK). All other chemicals and reagents used were of analytical grade.

### 3.2. Preparation of Chitosan Gel Formulations

The composition of OND gels to fabricate transdermal films are given in Table 4. The concentrations of the polymer (chitosan), plasticizer (Transcutol^®^ P) and terpenes (limonene, nerolidol, and eucalyptol) are determined in pre-formulation studies. 

**Table 4:** The codes and composition of chitosan based gel formulations of OND (w/w, % on wet basis).

Component	G-CONT	G-LIMO	G-NERO	G-EUCO
Chitosan	3	3	3	3
Lactic Acid	3	3	3	3
Limonene	---	1	---	---
Nerolidol	---	---	1	---
Eucalyptol	---	---	---	1
OND	0.5	0.5	0.5	0.5
Transcutol^®^ P	5	5	5	5
Tween 80	1	1	1	1
Deionized Water	qs. 100	qs. 100	qs. 100	qs. 100

Briefly, chitosan was dispersed at 3% concentration. A mixture of Tween 80 (1%) and terpene (1%, limonene, nerolidol or eucalyptol) was homogenized in water at 8000 rpm during 5 min. OND was dissolved in water and combined with the emulsion prepared. The OND loaded emulsion was added to the chitosan gel base and finally Transcutol^®^ P (5%) was added as a plasticizer agent and stirred continuously until a homogeneous gel was obtained. In order to evaluate the effects of penetration enhancers, a control formulation was also prepared following the same method and components without adding penetration enhancer (G-CONT).

### 3.3. Mechanical Properties of the Chitosan Gels-Texture Profile Analysis (TPA)

The mechanical properties of the chitosan gel formulations coded with G-LIMO, G-NERO and G-EUCO consist of different type of terpene as penetration enhancer were examined using texture profile analyzer (TPA, TA.XT Plus, Stable Micro Systems, Haslemere, Surrey, UK). Gel formulations were transferred into 20 mL volume glass beakers and were placed in the ultrasonic water bath for 20 min prior to experiments to remove any air bubbles. TPA was performed using TPA. After the temperature of gel formulations maintained at 37 ± 0.5 °C, the hemispherical analytical probe was twice compressed into each sample at a defined rate (2.0 mm·s^−1^) to a depth of 15 mm. The delay period between the end of the first and beginning of the second compression was 15 s. All analyses were performed six times and data collection and calculations were performed using the Texture Exponent 6.0.7.0 software package of the instrument. The resultant force-time plots were used for determination of the following mechanical properties: Hardness, compressibility, adhesiveness, cohesiveness and elasticity data evaluation was performed using the instrument’s Texture Exponent software package [28,47].

### 3.4. Formulation of OND Transdermal Films

The transdermal films of OND coded with T-CONT, T-LIMO, T-NERO, and T-EUCO were fabricated by casting of gel formulations mentioned in Table 4. Firstly, chitosan gels were prepared as mentioned above. Then, the gel formulations were sonicated to remove air bubbles, dropped into the petri dish (θ: 9.8 cm) and allowed to dry at 40 ± 2 °C for 24 h. Dried films were stored in a desiccator wrapped in aluminium foil. The transdermal films prepared were used in bioadhesion measurements and *in vitro* permeation studies within 24 h.

### 3.5. Physicochemical Characteristics of OND Transdermal Films

#### 3.5.1. Organoleptic Examination

Transdermal films of OND were evaluated by visual inspection in terms of color, transparency, smoothness, homogeneity and flexibility.

#### 3.5.2. Film Thickness

Film thickness of OND transdermal films was measured with a manual digital micrometer (QLR digit, IP4, PRC) places on each specimen. Mean values and standard deviations were calculated. 

#### 3.5.3. Uniformity of Weight

The uniformity of weight for each formulation was calculated in six pieces of 1.77 cm^2^ film by calculating their average weight, and the deviation from average weight was determined. 

#### 3.5.4. Uniformity of Drug Content

A known weight of film was dissolved and subsequently diluted with 0.9% (w/w) saline solution. Samples were filtered through membrane filters (0.45 µM, Millex LH, Billerica, MA, USA) prior to HPLC analysis. Each film formulation was tested in triplicate and the results were expressed as the mean and standard deviation.

### 3.6. In vitro Bioadhesion Test

The adhesive properties of the chitosan based transdermal films of OND (T-LIMO, T-NERO, and T-EUCO) and control formulation (T-CONT) prepared in this study was assessed on *in vitro* pig skin using the TA-XT Texture Analyser. Full thickness pig skin freed from visible hair with scissors and allowed to hydrate with 0.9% saline solution at 37 °C prior to the experiment. The skin was held on the lower platform of the texture analyser and the transdermal film was applied on it. The upper probe was immersed on the film surface (15 mm), kept in contact for 30 s, and then it moved at a constant speed of 1 mm·s^−1^. The force required to detach the film from the skin was determined as the peak value in resultant force-distance plot. The *in vitro* adhesion work in 1 cm^2^ was calculated with the following equation and the adhesion values were expressed as the mean of three replicates:

Bioadhesion work (mJ/cm^2^) = AUC_1-2_/πr^2^ (1)

where πr^2^ = Area of the transdermal formulation in contact with the skin; AUC_1-2_ = Area under the force-distance plot.

### 3.7. *In Vitro* Skin Permeation Study

#### 3.7.1. Skin Preparation

Full thickness skin was removed from the dorsal side of the freshly excised pig, were stored at −20 °C and used within 2 months of harvesting. On the day of the experiment, defrosted skin was freed from visible hair with scissors and dermatomed to a thickness of 750 µm, using a Zimmer Dermatome (Zimmer, Warsaw, IN, USA).

#### 3.7.2. Permeation Experiments

*In vitro* skin permeation of OND from chitosan based transdermal film formulations (T-CONT, T-LIMO, T-NERO, and T-EUCO) was investigated using Franz-type diffusion cells (Permegear, Hellertown, PA, USA) with an effective diffusion area of 1.77 cm^2^ and receptor volume of 12 mL. 0.9% saline solution was used as a receptor fluid to maintain sink conditions in the receiver compartment. The receptor medium was constantly stirred with a Teflon-coated magnetic stirrer at 500 rpm and thermostated at 37 ± 0.5 °C throughout the experiments. Dermatomed pig skin pieces were equilibrated in 0.9% saline solution for 30 min and then mounted on diffusion cells. A circular section (r = 1 cm) of transdermal film formulation was placed in the donor chamber onto the *stratum corneum* side of the skin, wetted with 100 µL of water and all cell donors were occluded with a Parafilm^TM^. 1 mL sample of the receptor was taken at predetermined time intervals over 24 h and replenished immediately with equal volume of the receptor phase. OND concentration was analysed by the HPLC method given below. Each experiment was performed at least three times.

#### 3.7.3. HPLC Analysis

The quantification of OND in this study was performed using the Shimadzu HPLC system (Shimadzu, Kyoto, Japan) which was equipped with a SPD-M20A UV/Vis detector, a LC-20AD pump and a reversed phase C18 column (250 mm × 4.6 mm; 5 µm, Waters Symmetry). The HPLC system was monitored by LC Solution Version (1.2.1) SP1 (Shimadzu) software. The mobile phase was a mixture of acetonitrile: phosphoric acid, (10 mmol) (16:84), filtered through membrane filters (0.45 µM, Millex LH) and eluted at a flow rate of 1 mL/min. Analyses were performed using a detection wavelength of 305 nm and a sharp peak were observed for OND at 2.8 min. The method was validated for selectivity, linearity, accuracy and precision. It was found to be linear over the concentration range 0.1–10 μg/mL with a high correlation coefficient (r^2^ > 0.999) and precise (intra and inter day variation < 2%) and accurate (recovery > 98%). There were no interfering peaks with OND confirming the selectivity of the method. Stability studies showed that OND was stable during 48 h in the mobile phase.

#### 3.7.4. Permeation Data Analysis

The *in vitro* skin permeation data obtained was graphically plotted as the cumulative amount of OND (μg/cm^2^) permeated into the receptor phase as a function of time. The permeation profiles provided the following parameters: The slope of the straight line portions of this plot yielded the values of flux *J_ss_* (μg/cm^2^/h). Enhancement ratio for flux (ER_flux_) was calculated according to the following equation: ER_flux_ = OND flux with enhancer in gel formulation/OND flux without enhancer in gel formulation (T-CONT).

### 3.8. ATR-FTIR Spectroscopy

Upon completion of the permeation experiment the receptor phases were removed and the diffusion cells were dismantled. The skin surface was washed in running water to remove the residual formulation and was gently blotted dry with soft tissue. The surface of the skin exposed to the donor compartment was punched out and vacuum dried. The skin pieces were placed *stratum corneum* side down onto the internal reflection element (ZnSe crystal having a trapezoidal cut of 45°) of an ATR-FTIR spectrometer (Perkin Elmer Spectrum 100 FT-IR Spectrometer, Wellesley, MA, USA). To ensure reproducible contact between the sample and the crystal, always the same pressure on top of samples was applied (force gauge 80 N). In order to minimize inter sample variation, the same piece of skin before treatment was used for normalization. The ATR-FTIR spectra were obtained in the frequency range of 4,000–650 cm^−1^ with a spectral resolution of 4 cm^−1^. The peak positions were assigned using Perkin Elmer Spectrum Version 6.0.2 software. Attention was focused on characterizing the occurrence of peaks near 2,920 and 2,850 cm^−1^ which were due to the asymmetric and symmetric CH_2_ stretching absorbances, respectively.

### 3.9. Statistical Data Analysis

Results are expressed as means of three to six experiments ± SD. Statistical data analyses were performed with the program GraphPadPrism5 while using *p* < 0.05 as a minimum level of significance in all cases. One way ANOVA, followed by the student-Newman-Keuls multiple comparison tests were performed. 

## 4. Conclusions

The data indicated that matrix type chitosan based transdermal films of OND could be prepared using with Transcutol^®^ as plasticizer. The chitosan gels used to fabricate transdermal films showed good mechanical properties and transdermal films prepared with these gels had good physicochemical properties and bioadhesiveness. The highest enhancement effect on *in vitro* permeation of OND across skin was observed with transdermal film formulations containing eucalyptol. That finding was confirmed with ATR-FTIR analysis results in which a more disordered *stratum corneum* lipid state in the presence of eucalyptol was shown.
